# Tannin-tolerant and Extracellular Tannase Producing *Bacillus* Isolated from Traditional Fermented Tea Leaves and Their Probiotic Functional Properties

**DOI:** 10.3390/foods9040490

**Published:** 2020-04-13

**Authors:** Kridsada Unban, Pratthana Kodchasee, Kalidas Shetty, Chartchai Khanongnuch

**Affiliations:** 1 Division of Biotechnology, School of Agro-Industry, Faculty of Agro-Industry, Chiang Mai University, Muang, Chiang Mai 50100, Thailand; kridsada_u@cmu.ac.th (K.U.); Pratthana.k@cmu.ac.th (P.K.); 2 Global Institute of Food Security and International Agriculture (GIFSIA), Department of Plant Sciences, North Dakota State University, Fargo, ND 58108, USA; Kalidas.Shetty@ndsu.edu; 3 Research Center for Multidisciplinary Approaches to Miang, Chiang Mai University, Chiang Mai 50200, Thailand

**Keywords:** *Bacillus*, tannin-tolerant, tannase, fermented tea, probiotic

## Abstract

A total of 117 *Bacillus* strains were isolated from Miang, a culture relevant fermented tea of northern Thailand. These strains were collected from 16 sampling sites in north Thailand. In this collection 95 isolates were tannin-tolerant *Bacillus* capable of growth on nutrient agar supplemented with 0.5% (*w*/*v*) total tannins from tea leaves extract (TE). The strains were also positive for pectinase, xylanase and amylase activity, while 91 and 86 isolates were positive for cellulase and β-mannanase, respectively. Only 21 isolates producing extracellular tannase were selected for further characterization. Identification by 16S rRNA gene sequence analysis revealed that more than 50% (11 of 21 isolates) were *Bacillus* *tequilensis*, whereas the remaining were *B. siamensis* (3), *B. megaterium* (3), *B. aryabhattai* (3) and *B. toyonensis* (1). *B. tequilensis* K34.2 produced the highest extracellular tannase activity of 0.60 U/mL after cultivation at 37 °C for 48 h. In addition, all 21 isolates were resistant to 0.3% (*w*/*v*) bile salt, sensitive to gentamicin, erythromycin, vancomycin and kanamycin and also tolerant to acidic condition. Cell hydrophobicity varied from 9.4 to 80.4% and neutralized culture supernatants of some *Bacillus* isolates showed bacteriocin producing potentiality against *Samonella enterica* serovar Typhimurium TISTR 292. All tested probiotic properties indicated that *B. tequilensis* K19.3, *B. tequilensis* K34.2 and *B. siamensis* K19.1 had high probiotic potential. This is the first report describing tannin-tolerant *Bacillus* and their extracellular tannase producing capability in Miang, a traditional fermented tea of Thailand.

## 1. Introduction

Fermented tea leaves, called “Miang”, is a traditional fermented food product made from *Camellia sinensis* var. *assamica* that has been important in the sociocultural lifestyle of northern Thailand for over several hundred years. A typical Miang production process mainly consists of fermentation of steamed tea leaves without adding other nutritional substances in a variety of containers such as bamboo basket or clay jar for several days or up to a year without the use of any preservatives. However, the steps of fermentation process of Miang are different depending on the ethnicity of the local producer [1]. Since tea leaves are rich in phenolic compounds, the microbial fermented tea develops unique functions and has multiple beneficial effects on human health. Previous investigation also confirmed that Miang contains similar bioactive compounds including polyphenols, flavonoids, catechins, caffeine, gallic acids, tannins, volatile flavor and aromatic compounds which result in the strong aromatic odor and taste in range of indigenous fermented teas [2,3,4,5,6]. Furthermore, number of studies have described bioactive benefits and human health-relevant effects of tea phenolic compounds, including reducing the risk of cardio vascular disease, improving oral hygiene, cancer prevention, reduction of cholesterol level, and modulating blood pressure [7,8,9,10]. Therefore, due to the potential for such health relevant bioactive metabolites, traditional Miang fermentation and its use as the functional food or nutraceuticals was proposed and targeted [1].

Like in other fermented foods, the key microorganisms which play an important role in Miang fermentation are lactic acid bacteria [11,12,13]. In the last two decades, *Lactobacillus pentosus*, *Lactobacillus vaccinostercus*, *Enterococcus casseliflavus*, *Enterococcus calmelliae*, *Lactobacillus thailandensis*, *Lactobacillus camelliae* and *Pediococcus siamensis* have been reported to be involved in Miang fermentation [14,15]. Chaikaew et al. [16] also reported that *L. plantarum* group was considered to be the predominant lactic acid bacteria in Miang. In addition to lactic acid bacteria, yeasts have also been reported to be involved in tea leaves fermentation [11]. Recently, *Candida ethanolica* has been reported to be the dominant species in 47 Miang samples from upper northern Thailand [13]. Since tea leaves contain significant amounts of phenolic compounds, especially tannins that are known to be microbial growth inhibitors, the ability of microorganisms associated with the tea leaves fermentation to survive under tannin-rich conditions are of interest [17]. Many studies have evaluated tannin-tolerant lactic acid bacteria that have been isolated from Miang [11,14,15,16]. Chaikaew et al. [16] also confirmed that 23 isolates from 311 lactic acid bacterial strains isolated from Miang samples belonged to *L. pentosus* and exhibited high tannin-tolerant capabilities in medium containing 2.5% (*w*/*v*) tannins. Moreover, tannin-tolerant yeasts in Miang have also been described, where *Candida ethanolica*, *Pichia manshurica* and *Pichia occidentalis* were confirmed for their tannin-tolerant capability when cultivated in medium containing high tannin up to 5% (*w*/*v*). Furthermore, most of the yeast isolates obtained from Miang were harboring both tannin-tolerant capability and tannase producing ability [13]. Previous reports on the flavor constituents of Miang have also revealed that Miang possesses larger amounts of phenolic compounds than steamed tea leaves [5]. The useful therapeutic phenolic compounds which are related to the catechins and tannins were also found in Miang at higher concentrations when compared to fresh tea leaves, particularly in Miang made from young tea leaves [6]. Recent studies have also indicated the presence of endospore forming bacteria, which was significantly detected from 40 Miang samples in high numbers ranging from 40% to 50% of total bacterial counts [18]. Further understanding of this endospore forming species diversity of *Bacillus* isolated from Miang and other fermented tea and their metabolic role is essential for understanding the functional health benefits.

Therefore, this study investigated the tannin-tolerant *Bacillus* isolated from Miang sourced from north Thailand and further characterized basic properties, such as tannin- and polysaccharide-degrading enzyme-producing capability. The extracellular tannase producing *Bacillus* isolates were also selectively identified and studied for their probiotic properties. The overall aim was to advance better understanding of the role of tannin tolerant *Bacillus* in the Miang fermentation process, as well as gain potential insights for long term functional applications in human health. 

## 2. Materials and Methods

### 2.1. Sampling and Isolation of Bacillus *spp.*

Miang samples were collected from 16 local markets of various locations in upper northern Thailand including Chiang Mai (15 samples), Chiang Rai (8 samples), Lampang (4 samples), Phayao (2 samples), Phrae (3 samples), and Nan Province (7 samples) (Table 1). All samples were transported to the laboratory in sterile polyethylene bags on ice and analyzed immediately. A sample of 10 g each was mixed well with 90 mL of sterile 0.85% (*w*/*v*) NaCl solution using Masticator Homogenizer (Basic/Panoramic, IUL, S.A., Barcelona, Spain) for 10 min. The sample was further diluted in a 10-fold dilution series and heated at 80 °C for 12 min. The suitable dilutions were spread plated onto nutrient agar (NA) medium (Merck, Darmstadt, Germany) and incubated aerobically at 37 °C for 24 h. The colonies showing different morphological characteristics were picked from the plates and purified by repeated streaking onto the same nutrient agar medium for further characterization. Stock cultures were kept in nutrient broth containing 25% (*v*/*v*) glycerol and stored at −80 °C.

### 2.2. Tannin-Tolerant Ability of Bacillus *spp*.

The tannin tolerance of isolated *Bacillus* spp. in tea leaves extract (TE) was evaluated on NA supplemented with 20% (*v*/*v*) crude TE. Crude TE was prepared from local Miang prepared from Assam tea sourced from Chiang Mai as described by Chaikaew et al. [16]. Briefly, fresh young tea leaves (1000 g) were washed with tap water and then steamed for 30 min. The steamed tea leaves were mixed with 3000 mL sterile water and the mixture was homogenized in a Masticator blender (Basic/Panoramic, IUL, S.A., Barcelona, Spain) for 5 min. The supernatant was collected after centrifugation at 6000 rpm for 20 min, and concentrated by EYELA N-1000 rotary evaporator (Tokyo Rika-kikai, Co. Ltd., Tokyo, Japan) at 40 °C for 12 h to obtain a final volume of 200 mL. The TE solution was estimated for total tannins content using analysis method for tannin in tea infusion with some modification based on method of Tabasum et al. [19]. A single colony of each *Bacillus* isolate was transferred to NA supplemented with TE to obtain the final concentrations of 0.5% (*w*/*v*) total tannins. The growth of bacterial isolates was observed after incubation at 37 °C for 48 h.

### 2.3. Test for Extracellular Tannase and Polysaccharide Degrading Enzymes Production

All tannin-tolerant *Bacillus* isolates were investigated for their capability for production of extracellular tannase and polysaccharide degrading enzymes. All isolates were transferred into NA media containing 0.5% (*w*/*v*) tannic acid and incubated at 37 °C for 48 h. Growth and clear zone surrounding colonies represented the extracellular tannase activity and were observed after adding 1% (*v*/*v*) FeCl_3_ solution [20]. Similar to tannase activity, 0.5% (*w*/*v*) of carboxymethyl cellulose (CMC), locust bean gum (LBG), soluble starch and pectin supplemented with 0.01% (*w*/*v*) trypan blue were independently used to detect extracellular activities of cellulase, β-mannanase, amylase and pectinase, respectively. The isolates producing extracellular tannase, distinguished from the clear zone formed surrounding the colony, were selected to determine enzyme activity in culture broth. This was done by taking 1% (*v*/*v*) overnight inoculum of each isolate and transferring into 100 mL of nutrient broth containing 0.5% (*w*/*v*) tannic acid, pH 6.5, and shaking at 100 rpm at 37 °C for 48 h. The culture broth sample was collected at 24 and 48 h and cell free supernatant (CFS) was separated by centrifugation 10,000× *g* at 4 °C for 10 min and used for determination of extracellular tannase activity using a modified method of Sharma et al. [21]. Briefly, 0.125 mL of proper dilution of crude enzyme was mixed with 0.125 mL of substrate (12.5 mM of methyl gallate in 0.05 M of citrate buffer, pH 5.0) and incubated at 37 °C for 10 min. The reaction mixture was then mixed with 0.15 mL of 0.67% (*w*/*v*) methanolic rhodanine and incubated at 37 °C for 5 min. Next, 0.1 mL of 0.5 N potassium hydroxide was added and incubated at 37 °C for 5 min. The reaction mixture was filled with 2.0 mL distilled water and absorbance was measured at 520 nm. One unit of tannase activity was defined as the amount of enzyme that liberated 1 μmole of gallic acid per minute under the assay condition.

### 2.4. Identification of Extracellular Tannase Producing Bacillus *spp.* and Phylogenetic Analysis

All extracellular tannase producing *Bacillus* spp. isolates were identified using molecular identification via 16S rRNA gene analysis. The genomic DNA of each isolate was extracted from the bacterial cell following the standard protocol described by Sambrook and Russell [22]. The 16S rDNA fragment was amplified by polymerase chain reaction (PCR) using genomic DNA as a template with bacterial universal primers, 27F (5′-AGA GTT TGA TCC TGG CTC AG-3′) and 1525R (5′-AAG GAG GTG WTC CAR CC-3′) [23]. The amplification reactions were carried out in a thermal cycler (MyCycler DNA thermal cycler; Bio-Rad, Hercules, CA, USA) following the method of Kim and Chun [24]. The 16S rRNA gene sequences were compared to other genes in the GenBank and EzBioCloud databases, and the phylogenetic tree was created based on the neighbor-joining method by MEGA version 4.0 software [25]. All 16S rRNA gene sequences generated in this study have been deposited in the NCBI GenBank database under accession number MH889120 to MH889140.

### 2.5. Probiotic Potential Assessment

#### 2.5.1. Acid and Bile Salt Tolerances

All 21 extracellular tannase producing *Bacillus* spp. were tested for their tolerance to acidic condition and bile salts. Tolerance to acidic condition was determined using the method of Argyri et al. [26] with some modification. Briefly, 5 mL of bacterial cells from overnight (18 h) cultures were harvested by centrifugation 10,000× *g* at 4 °C for 10 min, washed twice with PBS buffer (pH 7.2), and resuspended in 5 mL of the same buffer solution. This suspension was inoculated (1%, *v*/*v*) into 5 mL of PBS solution pH 7.2 (control) and the PBS solution was adjusted to pH 2.0 and 3.0 with hydrochloric acid (1 M). These treated suspensions were incubated at 37 °C for 3 h. Viable cell count was determined by plating on NA and incubated at 37 °C for 12 h. The viable cell count was expressed as log value of colony-forming units per mL (logCFU/mL). The survival percentage was calculated as follows: survival (%) = [final (logCFU/mL)/control (logCFU/mL)] × 100.

Bile salts tolerance of bacterial strain was determined according to the method of García-Hernández et al. [27]. Briefly, 5 mL of bacterial cell from overnight (18 h) cultures were harvested by centrifugation at 10,000× *g* at 4 °C for 10 min, washed twice with PBS buffer (pH 7.2), and resuspended in 5 mL of the same buffer solution. The cell suspension was inoculated (1%, *v*/*v*) into 5 mL of PBS solution supplemented with 0.3% (*w*/*v*) bile salts (HiMedia, Mumbai, India) and PBS without bile salts served as control, after which all tubes were incubated at 37 °C. Following 3 h of incubation, the viable cell count was determined, and survival rate was calculated.

#### 2.5.2. Cell Surface Hydrophobicity

The cell surface hydrophobicity of selected bacilli was determined in terms of the bacterial cell ability in adhering to hydrocarbons (MATS: Microbial Adhesion to Solvents), according to the methodology described by García-Hernández et al. [27]. Bacterial cultures in stationary phase were harvested by centrifugation 10,000× *g* at 4 °C for 10 min, washed twice with PBS buffer (pH 7.2), and resuspended in 5 mL of the same buffer solution. The bacterial concentration was adjusted with PBS to OD600 = 1 (A0), and then an equal volume of toluene (BDH Chemicals, Ltd., Poole, England) was added. Toluene was chosen as a nonpolar solvent because it reflects cell surface hydrophobicity and hydrophilicity [28]. The two-phase system was completely mixed for 5 min. After 1 h of incubation at 37 °C, the aqueous phase was measured again (A1). MATS percentage was calculated according to the following equation: MATS (%) = [(A0 − A1)/A0] × 100. Isolates with MATS above 50% were considered to be hydrophobic.

#### 2.5.3. Antibiotic Susceptibility Test

Antibiotic susceptibility of 21 extracellular tannase producing strains was determined by disk diffusion method according to the guidelines of the Clinical and Laboratory Standards Institute [29]. Antibiotic resistance of the isolates was tested against four selected antibiotics, including gentamycin (10 μg), erythromycin (15 μg), vancomycin (30 μg) and kanamycin (30 μg). Fifty microliters of the active bacterial suspension (10^5^–10^6^ CFU/mL) was spread evenly on the NA plate and antibiotics discs were placed on the plates. After 24 h of incubation at 37 °C, the inhibition zone diameters were measured including the diameter of the discs. Breakpoints for the interpretation of inhibition zone were expressed as sensitive, S; intermediate, I; and resistant, R as described by CLSI [29].

#### 2.5.4. Antimicrobial Activity

The antimicrobial activity of the isolated *Bacillus* strains was assessed against *Salmonella enterica* serovar Typhimurium TISTR 292 using the well diffusion assay according to Abid et al. [30] with slight modification. Briefly, 50 μL of pathogen cell (10^6^–10^7^ CFU/mL) were spread onto agar plates. Cell-free culture supernatants (CFCS) were collected by centrifugation (10,000× *g* at 4 °C for 15 min) and filtered through 0.22 μm membrane filter (Millipore, Bedford, MA, USA). To demonstrate the antimicrobial activity, 75 μL of pH neutralized CFCS (pH 6.5) was added to each well (cut with sterile 6 mm Cork borer) of the pathogen cells agar plates. The agar plates were incubated at 37 °C for 18 h. Growth inhibition was read by measuring the diameter of the inhibition zones. 

## 3. Results and Discussion

### 3.1. Isolation of Bacillus *spp.*

A total of 117 presumptive *Bacillus* isolates were obtained from the 39 samples of Miang collected from 16 sampling sites within six provinces of northern Thailand (Table 1). All *Bacillus* isolates clearly showed the properties of bacteria in the genus *Bacillus* such as Gram-positive, rod shape, endospore-forming ability and catalase positive. The largest numbers of 51 *Bacillus* isolates were confirmed from Chiang Mai province, whereas the smallest numbers of 7 isolates were confirmed in Miang collected from Phayao province. The high number of samples obtained from Chiang Mai province aligns with the high number of Miang plantation areas which are also well distributed in various districts [1]. Furthermore, the previous reports related to this fermented tea over recent decades mostly investigated samples from Chiang Mai area [5,11,31]. Nan and Phrae provinces also have a long history of Miang production and provide Miang product with unique characteristics that differs from Chiang Mai and Chiang Rai areas [1]. Until now, there have been few published reports on relevance of endospore-forming bacteria from Miang or other fermented tea leaves products. However, the number of endospore-forming bacteria which accounted for approximately 40–45% of total bacterial counts in 40 Miang samples collected from twenty producing locations were detected and therefore suggested that they play specific roles in Miang fermentation [18].

### 3.2. Tannin Tolerance of Bacillus *spp.*

**Figure 1:**
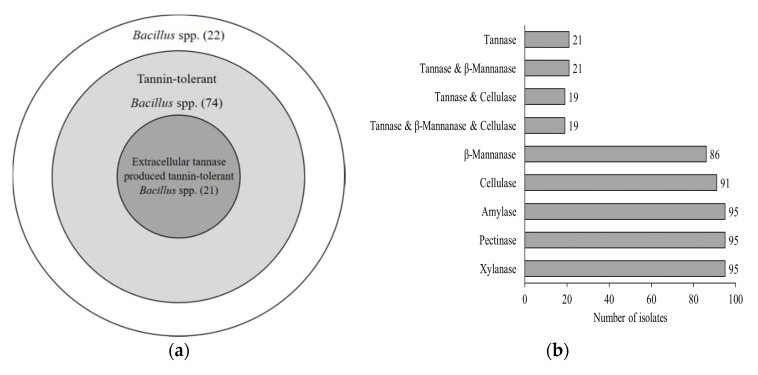
Venn diagram showing the number of *Bacillus* spp. isolated from Miang based on their tannin-tolerant characteristic (**a**) and a bar graph comparing the different extracellular enzyme characteristic of tannin-tolerant *Bacillus* spp. isolates (**b**).

**Figure 2:**
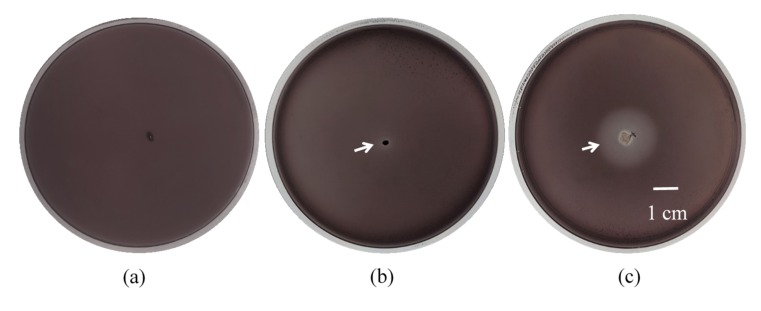
Clear zone formed on NA supplemented with 0.5% (*w*/*v*) of tannic acid by extracellular tannase produced from tannin-tolerant *Bacillus* spp. isolates incubated at 37 °C for 48 h. No clear zone formed (**a**), smallest clear zone formed (**b**), and widest clear zone formed (**c**). Arrows indicate clear zone formed.

Tannins concentration in TE prepared for this study was determined after product preparation and also prior to using in medium preparation. The TE from this experiment composed of approximately 2.5% (*w*/*v*) total tannins. All 117 *Bacillus* isolates were tested for their tolerance to tannins in TE at the final concentration of 0.5% (*w*/*v*) total tannins and it was found that 95 isolates or approximately 81% were tannin-tolerant based on their growth ability in NA supplemented with 0.5% (*w*/*v*) total tannins (Figure 1a). However, only 21 isolates formed clear zones surrounding the colonies in varying size implying that they were able to produce extracellular tannase. Among 95 isolates of tannin-tolerant *Bacillus*, 22% (21 of 95) exhibited the extracellular tannase producing ability. The widest clear zone (Figure 2c) was compared to the smallest (Figure 2b) and control or non-clear zone (Figure 2a). It is known that tea leaves contain significant amounts of phenolic compounds, particularly tannins and other tea phenolics, which have been reported to have inhibitory effect on growth of many microorganisms [32]. Therefore, the microorganisms capable of growth on this substrate potentially harbor the special metabolic mechanisms that allow tolerance to survive the toxicity of tea tannins via the induction of tannin degradation pathways [17]. Likewise, Zhao and Shah [33] also confirmed that the microorganisms originating from tannin-rich environments may have additional response mechanism to overcome the adverse effects of tannin stress to allow their metabolic activity and/or survival. However, 22 of the 117 *Bacillus* spp. isolated from Miang (18.8%) did not show any growth on NA supplemented with 0.5% (*w*/*v*) total tannins. This suggested that these 22 tannin-tolerant negative strains may harbor tannin-tolerant properties, but at concentrations lower than 0.5% (*w*/*v*) total tannins. Field and Lettinga [34] reported the toxicity of tannin against various microorganisms was approximately 0.3–0.7% (*w*/*v*) and showed 100% inhibition of *B. subtilis*.

### 3.3. Extracellular Polysaccharide Degrading Enzyme Production Test

In addition to the capability for survival in high-tannin condition, 95 tannin-tolerant *Bacillus* spp. were tested for their ability to produce extracellular polysaccharide degrading enzymes including amylase, pectinase, xylanase, cellulase and β-mannanase by observation of the clear zone formation in NA supplemented with the specific substrates as described previously. We have found that all tannin-tolerant *Bacillus* isolates were positive for pectinase, amylase and xylanase (Figure 1b). However, only 91 isolates (96%) were positive for cellulase production based on the visualization of the clear zone surrounding colonies on NA supplemented with CMC, while 4 isolates were negative. Similar to cellulase production, only 86 isolates (91%) were positive for β-mannanase, while 9 isolates were negative (Figure 1b). Furthermore, among the 21 extracellular tannase producers, 19 isolates were also found to form clear zones both on CMC and LBG and only 2 isolates (K22.1 and K23.1) were positive for β-mannanase. Based on the results from this study, most of tannin-tolerant *Bacillus* spp. were highly associated with the ability to produce extracellular polysaccharide degrading enzymes particularly pectinase, xylanase and amylase (100% positive) and 96 and 91% positive for cellulase and β-mannanase production, respectively. This indicates that the capability to produce extracellular polysaccharide degrading enzymes reflects the effort of the bacteria to find carbon source for their survival and extracellular cellulose, β-mannanase, pectinase, amylase and xylanase producing *Bacillus* strains have been well documented in several studies on fermented products [35,36,37]. Some of the extracellular polysaccharide degrading enzymes produced by tannin-tolerant *Bacillus* spp. mentioned in this study may be involved in the Miang fermentation process.

### 3.4. Identification of Tannase Producing Bacillus spp. and Phylogenetic Analysis

**Figure 3:**
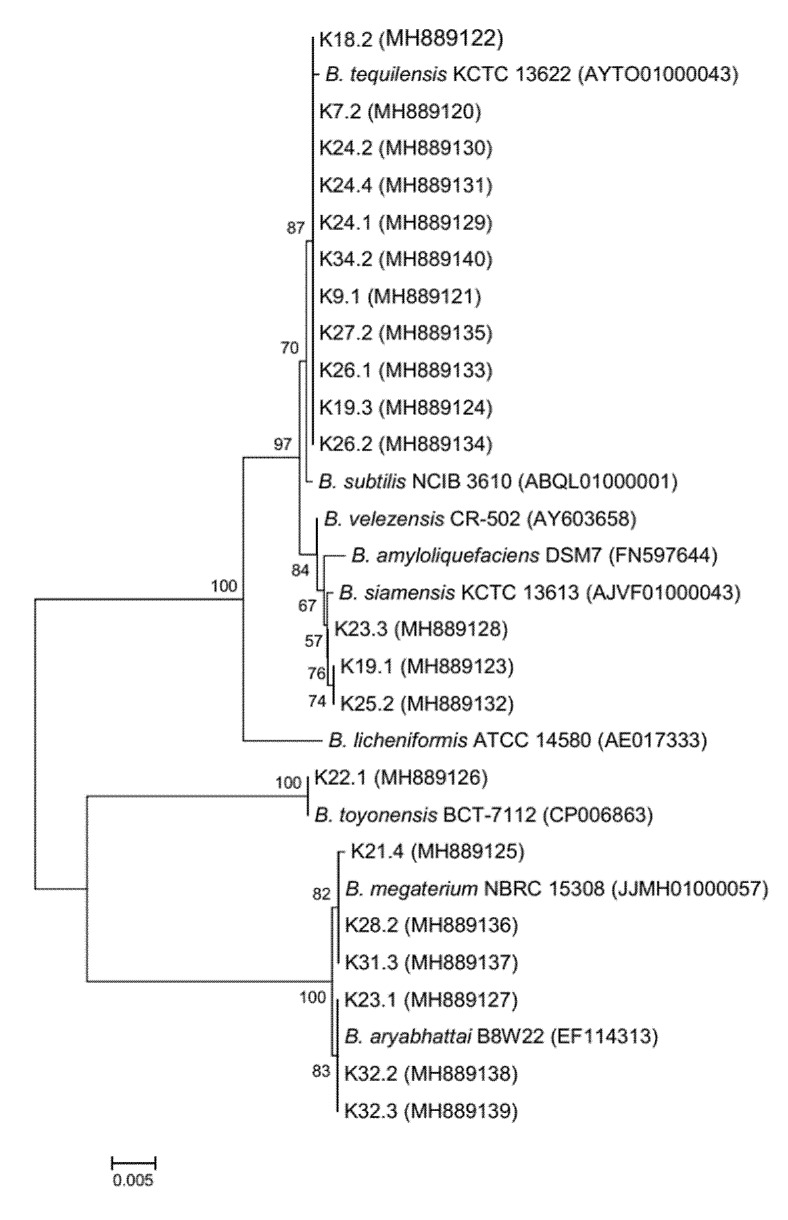
Neighbor-joining phylogenetic tree based on the 16S rRNA gene sequence of tannin-tolerant *Bacillus* spp. isolated from Miang and their related taxa.

Molecular identification techniques present an alternative to other conventional methods because the independent genome for the physiological characteristics may vary among the species. Moreover, the techniques are more reproducible, and the results are achieved more rapidly than with traditional methods. All tannase producing *Bacillus* isolates were identified using 16S rRNA gene analysis and the data are presented in Table 2. The majority of *Bacillus* (11 of 21 isolates) were identified as *Bacillus tequilensis* while the remaining as *B. siamensis* (3 isolates), *B. megaterium* (3 isolates), *B. aryabhattai* (3 isolates) and *B. toyonensis* (1 isolate), which showed similarities in 16S rRNA gene sequence related to their closest type strain in the narrow range between 99.8% and 100.0%. The phylogenetic trees based on the sequences of the 16S rRNA gene were constructed by the neighbor-joining method comparing between each *Bacillus* strain (Figure 3). From the results, most tannase producing *Bacillus* spp. isolated from Miang belong to species *B. tequilensis* which is closely related to *B. subtilis* [38]. A previous report found that *B. siamensis* and *B. licheniformis* have been isolated from tea leaves (raw material for Miang fermentation) and suggested involvement in Miang fermentation process [39].

### 3.5. Extracellular Tannase Production

**Figure 4:**
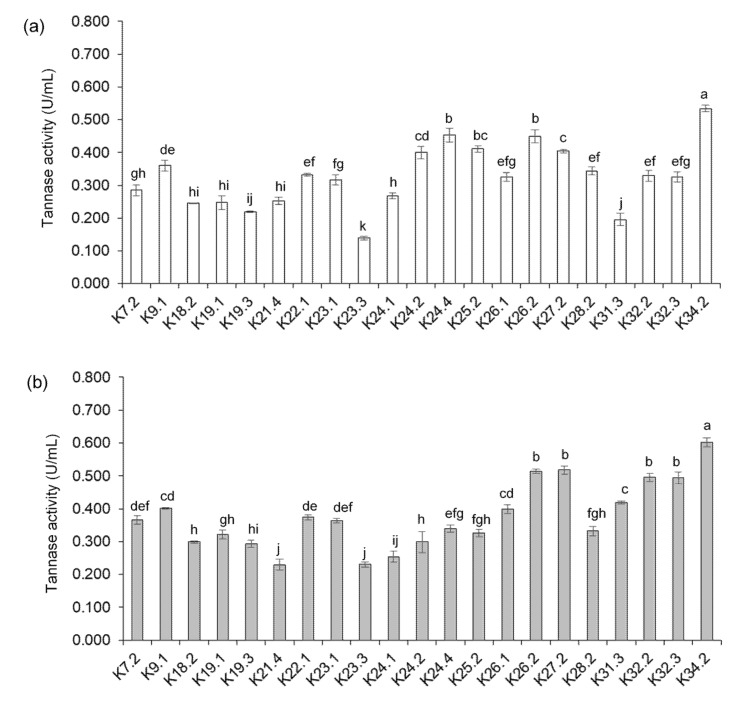
Tannase activity of 21 tannin-tolerant *Bacillus* spp. from culture broth containing 0.5% (*w*/*v*) tannic acid incubated at 37 °C for 24 h (**a**) and 48 h (**b**).

The tannase activity of all 21 isolates of extracellular tannase producing strains were determined from cell-free supernatant fraction (CFSF) separated from 24 and 48 h old culture broth containing 0.5% (*w*/*v*) tannic acid and the results are presented in Figure 4. Extracellular tannase were detected from CFSF of all isolates with variation in activity. *B. tequilensis* K34.2 showed the highest activity of 0.53 U/mL from 24 h to until the end of the experiment at 48 h (0.60 U/mL), while the lowest activity of 0.25 U/mL was observed in *B. megaterium* K21.4 and *B. siamensis* K23.3 at 48 h growth phase. Most of the reported bacterial tannase producers correspond to the presence of extracellular tannase [40]. Until now, several tannase producing *Bacillus* spp. have been identified such as *B. pumilus*, *B. polymyxa* [41], *B. licheniformis* [20,42], *B. cereus* [43], *B. sphaericus* [44], *B. massieliensis* [45], *B. subtilis* [46] and *B. gotthelii* [47]. This study is the first report on extracellular tannase produced by *B. tequilensis* and *B. siamensis*.

### 3.6. Assessment of Probiotic Potentiality of Tannin-Tolerant Bacillus *spp.*

A total of 21 tannin-tolerant *Bacillus* isolates were selected to determine probiotic potential and the results are shown in Table 3. Acid tolerance is generally considered as an essential assessment criterion for probiotic evaluation, since the strains have to survive the acidic condition of gastrointestinal tract environment [48,49]. The tolerances of all 21 isolates against low pH were examined by exposing the strains to pH 2.0 and 3.0 for 3 h. Most of the isolates were able to survive after being exposed to pH 3.0 for 3 h with varying degree of survival rate in the range of 51.74–99.30%. *B. siamensis* K25.2 showed excellent survival of 99.30%; however, *B. toyonensis* K22.1, *B. megaterium* K31.3, *B. aryabhattai* K32.2 and *B. aryabhattai* K32.3 were definitely inhibited by this acidic condition. When exposed to pH 2.0, only 13 isolates were able to tolerate acidic condition at pH 2.0, including 11 isolates of *B. tequilensis* (isolates K7.2, K9.1, K18.2, K19.3, K24.1, K24.2, K24.4, K26.1, K26.2, K27.2 and K34.2) and 2 isolates of *B. siamensis* (isolate K19.1 and K25.2). It was quite interesting to note that some *B. tequilensis* tolerated the acidic condition at pH 2 for 3 h with survival higher than 50% (isolates K7.2, K9.1, K18.2, K19.3, K27.2 and K34.2), whereas some strains were rather weak in acid tolerance (isolates K24.1, K24.2, K24.4, 26.1 and K26.2). Among them, *B. tequilensis* K19.3 exhibited most resistance to all tested acidic levels after 3 h at 75.24%. Our results are similar to the result of Parveen Rani et al. [50] who reported that *B. tequilensis* FR9 was able to survive after exposure to pH 2 and 3, with survival of 71.15% and 82.86%, respectively. Whereas, *Bacillus* sp. strain VS-5 was found to be the most resistant among the studied *Bacillus* species with survival of 98.78% after exposure to pH 1.2 for 3 h [51].

Tolerance to bile salt is considered to be vital characteristic for colonization and metabolic activity of bacteria in the intestinal tract and therefore it is an important characteristic of probiotic microorganisms [52]. The mean intestinal bile concentration is around 0.3% (*w*/*v*) and was selected for this study. All of the isolates were resistant to bile salt with varying degrees of resistance after 3 h exposure. *B. tequilensis* (isolates K7.2, K9.1, K18.2, K19.3, K24.1, K24.2, K24.4, K26.1, K27.2 and K34.2), *B. toyonensis* (isolate K22.1), *B. siamensis* (isolates K19.1, K23.3 and K25.2), and *B. megaterium* (isolate K21.4) were highly tolerant (survival more than 80%) to 0.3% bile salt. In comparison, *B. tequilensis* K24.1 was found to be the most resistant strain, with a survival of 98.65%, while *B. megaterium* K31.3 was found to be the least bile tolerant.

Another important criterion to select bacterial candidate for probiotic use is its ability to adhere to intestinal mucosal cells [53]. The isolates that showed a MATS value higher than 50% were *B. tequilensis* (isolates K7.2, K18.2, K19.3, K26.2, K27.2 and K34.2) and *B. siamensis* (isolates K19.1, K23.3 and K25.2). The high cell hydrophobicity were *B. tequilensis* K26.2 and *B. siamensis* K19.1 at 80.4 and 73.6%, respectively.

Previous studies suggested that candidate probiotic bacteria should not act as reservoirs for antibiotic resistance genes [54]. In this study, different functional types of antibiotics were tested as follows: cell wall inhibitors (vancomycin) and protein synthesis inhibitors (gentamicin, erythromycin and kanamycin). All the *Bacillus* isolates were found to be susceptible (data not shown) towards gentamicin and vancomycin that interrupt either protein synthesis or cell wall biosynthesis in the bacteria. At the same time, most of them were found to be susceptible toward erythromycin and kanamycin, but only *B. siamensis* K19.1 showed intermediate response to erythromycin and kanamycin. It should be noted that the sensitivity found in our study may be related to the concentration of each antibiotic and hence, several concentrations should be tested to confirm the results.

The capability to produce antimicrobial compounds is related to the metabolic products of bacteria such as organic acids, hydrogen peroxide, bacteriocins and short chain fatty acids, which are among the properties generally used to evaluate the probiotic potential of bacteria [30]. In this study, *S. enterica* were used as pathogenic indicator, where most of *Bacillus* isolates were able to show inhibition towards this pathogenic bacterium which included *B. tequilensis* (isolates K7.2, K9.1, K18.2, K19.3, K24.1, K24.2, K24.4, K26.1, K26.2, K27.2, K34.2), *B. siamensis* (isolates K19.1 and K25.2), *B. megaterium* (isolates K21.4, K28.2) and *B. aryabhattai* (isolates K23.1, K32.2, K32.3). The highest antimicrobial activities were from *B. tequilensis* isolates K7.2, K19.3 and K26.2.

## 4. Conclusions

In conclusion, *Bacillus* spp. diversity from traditional Miang, a fermented tea leaves product of northern Thailand was for the first time identified and characterized in this study. Among 117 *Bacillus* spp. isolated from Miang, 21 isolates showed the ability to produce extracellular tannase. The results of current study also showed that some tannin-tolerant *Bacillus* strains are potentially useful as probiotic bacteria. Some *B. tequilensis* and *B. siamensis* isolates showed high acid tolerance, bile salt tolerance, hydrophobicity and antimicrobial activity. Miang may also be considered a traditional fermented product with health-relevant functional benefits with antioxidant compounds such as high levels of phenolic acid and flavonoids [18], which are potentially biotransformed by tannase producing *Bacillus* spp. Moreover, due to the high numbers of *Bacillus* spp. in Miang, these bacteria in themselves may be considered as a possible probiotic. From the promising findings of this study more detailed analysis of probiotic potential of specific strains isolated from Miang should be undertaken. Further understanding of their health effects, including wider functional benefits of biotransformed tannin products should be investigated.

## Figures and Tables

**Table 1:** Number of Miang samples collected for isolation from upper northern Thailand.

Location	No. of Miang Samples Collected	No. of Strains Isolated
Chiang Mai Province		
San Kamphaeng	1	3
Doi Saket	4	14
Mae Taeng	5	17
Mae On	3	10
Phrao	1	5
Chiang Dao	1	2
Chiang Rai Province		
Thoeng	5	4
Wiang Pa Pao	3	8
Lampang Province		
Chae Hom	1	3
Mueang Pan	2	7
Mae Tha	1	3
Phayao Province		
Phu Sang	2	7
Phrae Province		
Mueang Phrae	3	9
Nan Province		
Song Khwae	3	11
Pua	2	7
Mueang	2	7
Total	39	117

**Table 2:** 16S rRNA gene sequencing result of 21 tannin-tolerant *Bacillus* spp. isolated from Miang.

Isolates	Closest Species	Similarity (%)	Length (bp)	Accession Number
K7.2	*Bacillus tequilensis* KCTC 13622(T)	99.93	1449	MH889120
K9.1	*Bacillus tequilensis* KCTC 13622(T)	99.93	1450	MH889121
K18.2	*Bacillus tequilensis* KCTC 13622(T)	99.93	1452	MH889122
K19.1	*Bacillus siamensis* KCTC 13613(T)	99.86	1448	MH889123
K19.3	*Bacillus tequilensis* KCTC 13622(T)	99.93	1447	MH889124
K21.4	*Bacillus megaterium* NBRC 15308(T)	100.00	1451	MH889125
K22.1	*Bacillus toyonensis* BCT-7112(T)	100.00	1458	MH889126
K23.1	*Bacillus aryabhattai* B8W22(T)	100.00	1457	MH889127
K23.3	*Bacillus siamensis* KCTC 13613(T)	99.93	1460	MH889128
K24.1	*Bacillus tequilensis* KCTC 13622(T)	99.93	1451	MH889129
K24.2	*Bacillus tequilensis* KCTC 13622(T)	99.93	1455	MH889130
K24.4	*Bacillus tequilensis* KCTC 13622(T)	99.93	1453	MH889131
K25.2	*Bacillus siamensis* KCTC 13613(T)	99.86	1454	MH889132
K26.1	*Bacillus tequilensis* KCTC 13622(T)	99.93	1446	MH889133
K26.2	*Bacillus tequilensis* KCTC 13622(T)	99.93	1449	MH889134
K27.2	*Bacillus tequilensis* KCTC 13622(T)	99.93	1454	MH889135
K28.2	*Bacillus megaterium* NBRC 15308(T)	100.00	1450	MH889136
K31.3	*Bacillus megaterium* NBRC 15308(T)	100.00	1452	MH889137
K32.2	*Bacillus aryabhattai* B8W22(T)	100.00	1451	MH889138
K32.3	*Bacillus aryabhattai* B8W22(T)	100.00	1456	MH889139
K34.2	*Bacillus tequilensis* KCTC 13622(T)	99.93	1454	MH889140

**Note:** “T ” is indicate the type strain of bacteria.

**Table 3:** Probiotic characteristics of tannin-tolerant *Bacillus* spp. strains.

Isolates	Survival Rate (%)			MATS (%)	Diameter Inhibition (mm) of *S. enterica*
	pH 2.0	pH 3.0	0.3% Bile Salt		
*B. tequilensis* K7.2	53.03 ± 1.51 ^bc^	77.47 ± 1.53 ^ef^	85.45 ± 1.16 ^def^	60.9 ± 2.6 ^cd^	14 ± 0.2 ^ab^
*B. tequilensis* K9.1	55.18 ± 1.40 ^bc^	88.55 ± 0.26 ^bc^	95.22 ± 1.22 ^abc^	49.2 ± 1.8 ^d^	7 ± 0.5 ^e^
*B. tequilensis* K18.2	51.77 ± 2.17 ^c^	85.11 ± 0.94 ^bcd^	82.55 ± 0.96 ^fg^	68.0 ± 1.3 ^bc^	12 ± 0.5 ^bc^
*B. siamensis* K19.1	73.77 ± 2.53 ^a^	83.04 ± 1.17 ^cde^	81.30 ± 1.57 ^fg^	73.6 ± 2.7 ^ab^	10 ± 0.1 ^c^
*B. tequilensis* K19.3	75.24 ± 1.63 ^a^	91.51 ± 1.85 ^b^	81.75 ± 1.55 ^fg^	54.2 ± 1.7 ^d^	16 ± 0.7 ^a^
*B. megaterium* K21.4	0.00	70.92 ± 1.69 ^fg^	83.78 ± 0.63 ^ef^	36.1 ± 1.8 ^e^	12 ± 0.8 ^bc^
*B. toyonensis* K22.1	0.00	0.00	89.67 ± 0.76 ^bcdef^	33.5 ± 1.5 ^e^	nd
*B. aryabhattai* K23.1	0.00	63.48 ± 1.45 ^hi^	74.48 ± 1.41 ^gh^	9.4 ± 2.4 ^g^	12 ± 0.6 ^bc^
*B. siamensis* K23.3	0.00	90.61 ± 1.1 ^b^	97.54 ± 2.25 ^ab^	61.5 ± 1.4 ^bcd^	nd
*B. tequilensis* K24.1	27.25 ± 1.24 ^d^	80.96 ± 1.08 ^de^	98.65 ± 0.67 ^a^	25.9 ± 2.9 ^ef^	8 ± 0.2 ^de^
*B. tequilensis* K24.2	16.33 ± 3.61 ^e^	60.83 ± 1.20 ^i^	88.86 ± 1.97 ^cdef^	30.5 ± 1.5 ^e^	7 ± 0.3 ^e^
*B. tequilensis* K24.4	13.52 ± 2.11 ^e^	65.74 ± 1.60 ^ghi^	91.46 ± 1.25 ^abcde^	33.0 ± 3.3 ^e^	9 ± 0.5 ^de^
*B. siamensis* K25.2	55.76 ± 1.53 ^bc^	99.30 ± 2.03 ^a^	92.93 ± 1.19 ^abcd^	59.7 ± 2.2 ^cd^	11 ± 0.9 ^c^
*B. tequilensis* K26.1	49.35 ± 1.63 ^c^	84.65 ± 1.99 ^bcde^	97.10 ± 1.48 ^abc^	35.1 ± 3.2 ^e^	13 ± 0.8 ^bc^
*B. tequilensis* K26.2	23.44 ± 1.77 ^de^	68.61 ± 1.79 ^gh^	69.15 ± 1.64 ^hi^	80.4 ± 1.2 ^a^	14 ± 0.8 ^ab^
*B. tequilensis* K27.2	51.74 ± 2.98 ^c^	89.59 ± 0.43 ^bc^	89.04 ± 2.37 ^bcdef^	59.0 ± 1.7 ^cd^	7 ± 0.4 ^e^
*B. megaterium* K28.2	0.00	51.74 ± 1.46 ^j^	73.92 ± 1.63 ^gh^	14.9 ± 1.3 ^fg^	12 ± 0.6 ^bc^
*B. megaterium* K31.3	0.00	0.00	59.62 ± 2.58 ^j^	30.5 ± 2.7 ^e^	nd
*B. aryabhattai* K32.2	0.00	0.00	64.59 ± 1.92 ^ij^	16.0 ± 1.5 ^fg^	10 ± 0.3 ^c^
*B. aryabhattai* K32.3	0.00	0.00	70.99 ± 0.40 ^hi^	28.6 ± 1.9 ^e^	11 ± 0.5 ^c^
*B. tequilensis* K34.2	62.97 ± 1.30 ^b^	85.23 ± 1.07 ^bcd^	96.30 ± 0.66 ^abc^	52.9 ± 2.8 ^d^	13 ± 0.4 ^bc^

**Note:** Means in row with different superscripts are statistically different at *p* < 0.05; MATS: microbial adhesion to solvents; nd: no inhibition zone detected.
